# Innovative Drugs to Target Renal Inflammation in Sepsis: Alkaline Phosphatase

**DOI:** 10.3389/fphar.2019.00919

**Published:** 2019-08-23

**Authors:** Femke Hümmeke-Oppers, Pleun Hemelaar, Peter Pickkers

**Affiliations:** ^1^Department of Intensive Care Medicine, Radboud University Medical Center, Nijmegen, Netherlands; ^2^Radboud Center for Infectious Diseases (RCI), Radboud University Medical Center, Nijmegen, Netherlands

**Keywords:** acute kidney injury, recombinant alkaline phosphatase, sepsis, septic shock, sepsis-associated acute kidney injury, chronic kidney disease, renal function, fibrosis

## Abstract

Sepsis-related mortality roughly doubles when acute kidney injury (AKI) occurs and end-stage renal disease is more common in sepsis-associated AKI survivors. So far, no licensed treatment for the prevention of AKI is available, however the data on alkaline phosphatase (AP) is promising and might change this. Sepsis-associated AKI is believed to be the result of inflammation and hypoxia combined. Systemic inflammation started by recognition of ‘pathogen-associated molecular patterns’ (PAMPs) such as lipopolysaccharide (LPS) which binds to Toll-like receptor 4 and leads to the production of inflammatory mediators. Due to this inflammatory process renal microcirculation gets impaired leading to hypoxia resulting in cell damage or cell death. In the process of cell damage so called ‘danger-associated molecular patterns’ (DAMPs) are released resulting in a sustained inflammatory effect. Apart from the systemic inflammation DAMPs and PAMPs also interact with receptors in the proximal tubule of the kidney causing a local inflammatory response leading to leukocyte infiltration and tubular lesions, combined with renal cell apoptosis and ultimately to AKI. In the longer-term, inflammation-mediated inadequate repair mechanism may lead to fibrosis and development of chronic kidney disease. AP is an endogenous enzyme that dephosphorylates and thereby detoxifies several compounds, including LPS. A small phase 2 clinical trial in sepsis patients showed that urinary excretion of tubular injury markers was attenuated and creatinine clearance improved in sepsis patients treated with AP. This renal protective effect was confirmed in a second small clinical phase 2 trial in sepsis patients with AKI. Subsequently, a large trial in sepsis patients with AKI was conducted using a human recombinant AP. In 301 patients no improvement of kidney function within 7 days after enrolment was observed, but kidney function was significantly better on day 21 and day 28 and all-cause 28-day mortality was significantly lower (14.4% in AP group versus 26.7% in the placebo group). Possible explanations of this lack of short-term kidney function improvement are discussed and potential effects of AP on renal repair mechanisms, including inflammation-mediated induction of fibrosis, that may explain the beneficial longer-term effects of AP are proposed.

## Background

Acute kidney injury (AKI) is defined by an increase in serum creatinine or a decrease in urine output (Kidney Disease: Improving Global Outcomes (KDIGO) Acute Kidney Injury Work Group, 2012), as representatives of loss of kidney function. The incidence of AKI in the intensive care unit (ICU) is up to 60% (Hoste et al., 2015) with sepsis as the most common cause of AKI, accounting for over 40% of cases (Angus et al., 2001). Sepsis-related morbidity, mortality, and costs of treatment represent a persistently high burden (Kumar et al., 2011; Fleischmann et al., 2016). Approximately half of sepsis patients need to be admitted to the ICU due to reduced or complete loss of function of one or multiple vital organs (Angus et al., 2001). Sepsis-related mortality is significant as one in three patients dies, and this roughly doubles when loss of kidney function occurs (Hoste et al., 2015). Patients that survive an episode of sepsis-associated AKI (SA-AKI) demonstrate an increased risk of developing end-stage renal disease later in life (Chawla et al., 2011).

SA-AKI was initially thought to result from a deterioration in macrohemodynamics, resulting in reduced kidney perfusion, ischemia, and subsequent tubulus necrosis (Uchino et al., 2005). However, AKI may occur during a state of increased renal blood flow, and histological findings do not show widespread tubular necrosis compatible with ischemia but instead a heterogeneous and patchy pattern of tubular cell injury (Langenberg et al., 2005; Langenberg et al., 2006). It has become increasingly clear that the pathogenesis of SA-AKI is a complex and multifactorial syndrome consisting of damage due to inflammatory processes, ischemic damage due to altered microcirculation, and impaired renal bioenergetics (Gomez et al., 2014; Bellomo et al., 2017).

The inflammatory response to a pathogen starts with the recognition of distinct “pathogen-associated molecular patterns” (PAMPs), by the innate immune system (Kawai and Akira, 2007). Through different signaling pathways, e.g., Toll-like receptors (TLRs), an inflammatory response is triggered causing the release of several inflammatory mediators. Subsequent cellular injury may lead to the release of adenosine triphosphate (ATP) (Eltzschig et al., 2012), that in turn acts as a “danger-associated molecular pattern” (DAMP). While the inflammatory response is essential in fighting an infection, during sepsis, this inflammatory response becomes unbalanced causing adverse and harmful effects (Chen and Nuñez, 2010; Gustot, 2011). Lipopolysaccharide (LPS) is part of the outer cell membrane of Gram-negative bacteria (Cohen, 2002) and is the most extensively studied PAMP. LPS binds to Toll-like receptor 4 (TLR4) which initiates the systemic inflammatory response (Peters et al., 2014a). Intriguingly, not only immune cells but also tubular cells express TLRs. Because LPS, among other PAMPs, DAMPs and inflammatory mediators, is filtered in the glomeruli, it can directly bind to TLR4 in the proximal tubule of the kidney (El-Achkar et al., 2006), within minutes after infusion (Good et al., 2009) causing activation of the complement system, coagulatory pathways, as well as release of cellular ATP, cytokines, and chemokines, transmigration of leucocytes, production of reactive oxygen species (ROS), and reactive nitrogen species (RNS) in the kidney (Peters et al., 2014a). Acute tubular lesions, apoptosis, and leucocyte infiltrates ultimately cause renal function impairment; AKI has occurred (Peters et al., 2014a).

Once the acute phase has passed, a phase of repair and regeneration often allows reversal of loss of kidney function to a great extent (Rabb et al., 2016). Some parts of the immune system that caused the initial damage play a role in the organ recovery as well; for example, the role of macrophages changes from proinflammatory to proregeneration (Jang and Rabb, 2014), also mediated through TLR4 receptor activation. When TLR4 on macrophages or interstitial dendritic cells are activated, it stimulates the production of interleukin-22 (IL-22), accelerating the reepithelialization by the tubular epithelial cells that have survived the first phase of acute injury (Kulkarni et al., 2014). An alternative response following organ damage is the formation of fibrosis; a deviant form of healing. Fibrosis originates from the production of myofibroblast cells and overproduction of extra cellular matrix by fibroblasts and mesangial cells (Anders and Schaefer, 2014). Unfortunately, these cells can also be activated by PAMPs and DAMPs *via* their TLRs. TLR activation by DAMPs on fibroblasts and mesangial cells leads to increased excretion of extracellular matrix, increased proliferation, and increased transition into myofibroblast cells (Anders and Schaefer, 2014).

In conclusion PAMPs and DAMPs exert proinflammatory effects systemically, but also locally in the kidney, leading to undesirable consequences. Nevertheless, the TLR-pathway also plays a role in the maladaptive regeneration following this initial response, leading to a profibrotic effect which is undesirable during healing. The finding that TLR activation plays a role in the initiation of regenerative effects, implicates that an immunomodulating treatment against sepsis which might interfere with PAMP/DAMP-TLR activation pathways is highly complex.

Up to now, no drug is licensed for the prevention and treatment of SA-AKI. Currently, the clinical focus is mainly on supportive care, such as renal replacement therapy, and secondary prevention to prevent further harm. Due to the much higher mortality of sepsis when accompanied by AKI, and the long-term impact on renal function after surviving the disease, the need for a treatment of SA-AKI is paramount.

## Alkaline Phosphatase

Alkaline phosphatase (AP) is an endogenous occurring detoxifying enzyme which is present throughout the body in four different iso-enzymes: germ-cell AP, intestinal AP, placental AP, and a non-tissue-specific form which is mostly derived from the kidneys, liver, and skeleton (Millán, 2006). As described above, for example the DAMP endotoxin contains two phosphate groups and removal of one of the phosphate groups by a dephosphorylating molecule results in a nontoxic lipid A moiety (Bentala et al., 2002). The dephosphorylated endotoxin can still bind to TLR4 but fails to activate it, and thus exerts TLR4-antagonistic properties. AP is able to dephosphorylate endotoxin (Poelstra et al., 1997). The presence of free phosphate, using enzymatic *in vitro* assays and *in vivo* histochemical analysis, revealing phosphatase activity present at the tubular brush borders (Poelstra et al., 1997; Bentala et al., 2002; Verweij et al., 2004) confirms this ability of AP and in accordance protects animals in Gram-negative *Escherichia coli* sepsis models, whereas not affecting survival when exposed to Gram-positive bacterium *Staphylococcus aureus* (Poelstra et al., 1997). By its dephosphorylating ability, AP is able to detoxify not only endotoxin, but various compounds. The widespread presence of AP indicates a wide range of functions during sickness and health. AP measured in serum is currently used as diagnostic tool in liver disease (Siddique and Kowdley, 2012), bone diseases (Ross et al., 2000), and testicular cancer (Neumann et al., 2011). However, AP might also play an important role in the treatment of the critically ill. Its dephosphorylating capacity could counteract the undesirable cascades of molecules including PAMPs (Koyama et al., 2002) and DAMPs (Picher et al., 2003) during sepsis. The lipid A part of the outer membrane of LPS contains two phosphate groups, of which one that can be dephosphorylated by AP (Koyama et al., 2002; Peters et al., 2014a). With its capability to detoxify LPS, AP was initially thought of to represent a promising antisepsis drug.

In addition to the detoxifying effect of AP on LPS, a potential other mode of action of AP during sepsis is the dephosphorylation of ATP. ATP is, under normal circumstances, located intracellular where it has an energy providing function. Cellular stress, caused by, e.g., hypoxia or inflammation, may cause ATP to be released into the extracellular space where it acts as a DAMP potentiating the inflammatory process (Bours et al., 2006). AP catalyses the dephosphorylation of extracellular ATP to adenosine diphosphate, adenosine monophosphate, and finally to adenosine (Peters et al., 2014a). Binding to its receptors in the nephron, adenosine may play a role in the regulation of renal blood flow by influencing vascular tone as well as regulating the glomerular filtration rate through tubuloglomerular feedback and renine release (Vallon et al., 2006). In addition, the adenosine receptors in the nephron are also present on immune cells (Ramakers et al., 2011) and when activated, induce protective effects on tissue by an anti-inflammatory response (Bauerle et al., 2011). Several other interventions related to the LPS–TLR4 pathway have been tested, including LPS antibodies and TLR4 antagonists. For example a large clinical study investigating the effects of a synthetic lipid A antagonist that blocks endotoxin-binding to the MD2–TLR4 receptor on mortality did not show a clinical benefit (Opal et al., 2013). Of interest, patients with Gram-positive bacterial infections appeared to do worse in the treatment group compared to placebo and it was suggested that comedication interfering with TLR4 signaling may have influenced the results. Of interest, the described unspecific effects of AP, removing phosphate groups from different compounds, may account for its therapeutic efficacy compared to much more specific interventions.

### Preclinical Data

Administration of exogenous AP has been extensively investigated in animal models, showing the capacity to significantly attenuate the plasma tumor necrosis factor-α (TNF-α) levels (van Veen et al., 2005), plasma IL-6 levels (van Veen et al., 2005), serum nitric oxide (NO) levels (Verweij et al., 2004), associated with less pronounced organ dysfunction (van Veen et al., 2005), and a significant increase in survival in various sepsis models (Bentala et al., 2002; Beumer et al., 2003; Verweij et al., 2004; Su et al., 2006) (see **Table 1**). A deeper analysis of the preclinical data has previously been published by Peters et al., (2013).

**Table 1:** Overview of alkaline phosphatase related (pre)clinical research.

AP related effects					
Study	Species	Model	Treatment	Sample size	Outcome
Yao et al. (1994)	Rats	IV LPS injection	Pretreatment with monophosphoryl lipid A (MLA; a dephosphorylated form of lipid A in LPS) 5 mg/kg for 24	NR	LPS: 89% mortality at 48 following administration LPS + MLA: 0% mortality at 48 following administration and inhibited all of the manifestations of disseminated intravascular coagulation produced by endotoxin.
Poelstra et al. (1997)	Rats	Intraperitoneal *E. coli*/*Staphylococcus aureus* injection	Levamisole (inhibitor of intestinal AP) 50 mg/kg BW	42	Inhibition of endogenous AP by levamisole significantly reduced survival of rats intraperitoneally injected with *E. coli* bacteria.
Preclinical					
Study	Species	Model	Treatment	Sample size	Outcome
Bentala et al. (2002)	Mice	Intraperitoneal D-galactosamine + LPS injection	IV bolus injection of 0.1 U placental AP	7	Survival rate: 100% (treated) vs 57% (untreated), P = NR
Koyama et al. (2002)	Rats	Oral LPS administration	Oral administration of 40 mg/kg BW l-phenylalanine (inhibitor of intestinal AP)	3	Serum LPS levels: 180 pg/ml (treated) vs 340 pg/ml (untreated), P < 0.05
Beumer et al. (2003)	Mice	Intraperitoneal *E. coli* injection	IV bolus injection of 1.5 U biAP	5	Survival rate: 80% (treated) vs 20% (untreated), P < 0.01 Body temperature decreased to: 36.2 ± 0.7°C (treated) vs 34.2 ± 0.8°C (untreated), P < 0.05
Beumer et al. (2003)	Piglets	IV LPS injection	IV injection of 2,500 U biAP	1–3 per group	Platelet counts decreased to: 41 ± 4 × 10^9^/L (treated) vs 19 ± 1 × 10^9^/L (untreated), P < 0.05
Verweij et al. (2004)	Mice	Intraperitoneal *E. coli* injection	IV bolus injection of 1.5 U placental AP	14	Survival rate: 100% (treated) vs 58% (untreated) P < 0.01 Serum NO levels increased to: 75 μmol/L (*E. coli*, untreated) vs 16 μmol/L [control (no *E. coli*)], P < 0.01; 39 μmol/L (*E. coli*, treated) vs 16 μmol/L [control (no *E. coli*)], P = NS.
van Veen et al. (2005)	Mice	Cecal ligation and puncture	IV bolus injection of 0.15 U/g BW biAP	8	Significant reduced systemic inflammatory response defined as lower peak-plasma levels of TNF-α, IL-6 an MCP-1 and significant signs of reduced injury to liver an lung defined as reduced serum AST/ALT levels and reduced MPO activity in the lung (myeloperoxidase, indicator for tissue inflammation). No significant improvement of survival was shown.
Su et al. (2006)	Sheep	Intraperitoneal feces injection	IV bolus injection of 60 U/kg BW Intestinal AP followed by continuous infusion of 20 U/kg/h for 15 h	NR	Median survival significantly improved following AP administration (23.3 vs 17, p < 0.05). Plasma IL-6 level: 0.16 AU (treated) vs 0.20 AU (untreated), P < 0.05 Gas exchange (PaO_2_:FiO_2_ ratio) decreased to: 320 mm Hg (treated) vs 50 mm Hg (untreated), P < 0.05.
Peters et al. (2015)	Rats	IV LPS injection	IV bolus injection of 1000 U/kg BW human recombinant AP (recAP)	18	*In vivo*: LPS administration significantly prolonged FITC-sinistrin half-life (as a measure of glomerular filtration fraction), which was prevented by recAP coadministration. RecAP prevented LPS-induced increase in proximal tubule injury markers. *In vitro*: LPS-induced production of TNF-α, IL-6, and IL-8 was significantly attenuated by recAP. This effect was linked to dephosphorylation, as enzymatically inactive recAP had no effect on LPS-induced cytokine production. RecAP-mediated protection resulted in increased adenosine levels through dephosphorylation of LPS-induced extracellular ATP and ADP. Also, recAP attenuated LPS-induced increased expression of adenosine A2A receptor.
Peters et al. (2016)	Rats	Renal ischemia (30 min) and reperfusion	IV infusion of 1,000 U/kg recAP	18	RecAP prevented I/R-induced alterations of renal hemodynamics immediately following reperfusion. RecAP treatment prevented I/R injury-induced renal inflammation.
Peters et al. (2016)	Rats	LPS infusion (30 min)	IV infusion of 1,000 U/kg recAP	18	LPS-induced systemic hemodynamic instability and impaired renal oxygenation was not influenced by recAP. RecAP attenuated biomarkers of renal inflammation and damage during endotoxin-induced shock.
Clinical					
Study	Design		Treatment	Sample size	Outcome
Heemskerk et al. (2009)	Multicenter double-blind, randomized, placebo-controlled phase IIa study		Bovine AP: IV bolus injection of 67.5 U/kg BW, followed by continuous infusion of 132.5 U/kg or placebo	36	In sepsis patients, median creatinine clearance [IQR] increased from 54 [24–84] to 76 [25–101] ml/min in the 24 h after AP treatment, while it decreased from 80 [77–91] to 59 [45–59] in the placebo group, P < 0.05. Pathophysiology of NO production and subsequent renal damage were assessed in a subgroup of 15 patients. A 42-fold induction (vs. healthy subjects) in renal inducible NO synthase expression was reduced by 80 ± 5% after AP treatment. In AP-treated patients, the increase in cumulative urinary NO metabolite excretion was attenuated, whereas the opposite occurred after placebo. Reduced excretion of NO metabolites correlated with the proximal tubule injury marker glutathione S-transferase A1-1 in urine, which decreased by 70 [50–80]% in AP-treated patients compared with an increase by 200 [45–525]% in placebo-treated patients.
Pickkers et al. (2012)	International double-blind, randomized, placebo-controlled phase IIa study		Bovine AP: IV bolus injection of 67.5 U/kg BW, followed by continuous infusion of 132.5 U/kg/24h or placebo	36	In patients with SA-AKI, there was a significant (P = 0.02) difference in favor of AP treatment relative to controls for the primary outcome variable (progress in renal function variables (ECC, requirement and duration of renal replacement therapy) in 28 days). The improvement of ECC was significantly more pronounced in the treated group relative to placebo (from 50 ± 27 to 108 ± 73 ml/min) for the AP group; and from 40 ± 37 to 65 ± 30 ml/min for placebo; P = 0.01). Reductions in RRT requirement and duration were not significantly different between groups. The results in renal parameters were supported by significantly more pronounced reductions in the systemic markers C-reactive protein, IL-6, LPS-binding protein, and in the urinary excretion of KIM-1 and IL-18 in AP-treated patients relative to placebo.
Pickkers et al. (2018)	International double-blind, randomized, placebo-controlled, dose-finding, adaptive phase IIa/b study		Human recombinant AP: 0.4 mg/kg (n = 31), 0.8 mg/kg (n = 32), or 1.6 mg/kg (n = 111) or placebo (n = 116), once daily for 3 days	301	In patients with SA-AKI, from day 1 to day 7, median ECC increased with 37.4 [26–65.4] and 26 [35.9–61.9] ml/min in recAP and placebo, P = NS. Improvement in ECC over 28 days was significantly better in the recAP group compared to placebo (P = 0.04). Day 28 and day 90 mortality for recAP and placebo were 14.4 and 26.7% (P = 0.02) and 17.1 and 29.1% (P = 0.03), respectively.

### Clinical Data

Bovine intestinal AP (biAP) was administered to 67 healthy volunteers in various doses to determine safety and pharmacokinetics (Pickkers et al., 2009). A proportional and linear response to the different loading doses was found. Continuous infusion of biAP for 24–72 h showed a stable level throughout the period of infusion and was used to determine the elimination half-life of approximately 8 h.

Following the efficacy studies in animals and the safety and kinetics studies in healthy volunteers, a small phase 2 clinical trial in 36 suspected or proven Gram-negative sepsis patients (with or without AKI) was conducted (Heemskerk et al., 2009). Patients received a loading dose of 67.5U/kg biAP and a continuous infusion of 132.5U/kg/24 h biAP for 24 h. No safety concerns emerged in this critically ill population. Overall, no signal that bovine AP affected the systemic inflammatory response or clinical outcome was observed in this small phase 2 trial. However, in patients treated with biAP an improvement of endogenous creatinine clearance (ECC) was observed, while renal function further deteriorated in patients treated with placebo. Of interest, the urinary excretion of tubular injury markers was less pronounced in the biAP-treated group compared to placebo. When looking at the subgroup of patients who had AKI at enrolment, the trial suggested better survival rates for the biAP-treated group compared to the placebo group (27% versus 60% respectively, p = 0.21), but obviously the trial was grossly underpowered to demonstrate clinically relevant effects.

In view of the renal effects of biAP, a second small phase 2 trial was designed to focus specifically on sepsis patients with signs of early AKI. This second phase 2 trial also included 36 patients with proven or suspected Gram-negative sepsis or shock and as an additional inclusion criteria proven AKI (as defined by AKIN criteria or rise in serum creatinine to >150µmol/l in the last 48 hours). The patients were treated with the same loading dose of biAP of 67.5U/kg followed by continuous infusion of biAP of 132.5U/kg/24 for 48 h. The renal protective effects of biAP were confirmed, as patients treated with biAP demonstrated a more pronounced improvement of renal function compared to patients treated with placebo (Pickkers et al., 2012).

**Figure 1:**
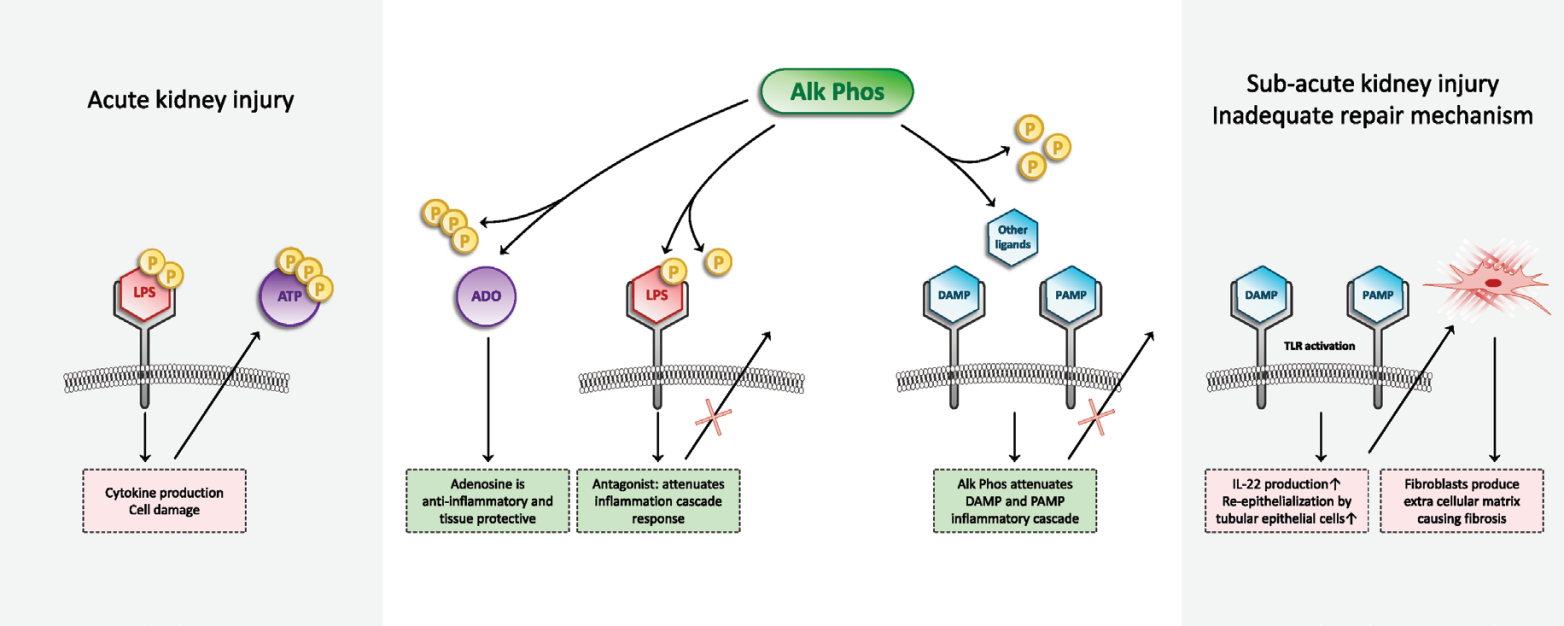
Schematic overview of the mechanism of action of recombinant AP. AP is able to dephosphorylate different compounds and thereby detoxifies these compounds. Left part of figure: Circulating endotoxin (LPS) in sepsis patients is filtered in the kidneys and recognized by tubular epithelial cells that express pathogen pattern receptors, such as TLR4. It is now recognized that the subsequent renal inflammatory response can be detrimental to kidney function. LPS contains two phosphate groups. RecAP is able to remove a phosphate group from LPS, after which the LPS can still bind to the TLR4, but does no longer induce the inflammatory cascade. Even more so, dephosphorylated LPS acts as a TLR4 antagonist for intact LPS containing two phosphate groups. During sepsis, ATP is released from cells. Extracellular ATP exerts proinflammatory effects. In addition to LPS as a PAMP, ATP acts as a DAMP. Dephosphorylation of ATP by recAP results in the formation of ADP, AMP, and eventually adenosine. Adenosine exerts anti-inflammatory and tissue-protective effects. Right part of figure: Apart from these mechanisms during acute inflammation, also kidney (maladaptive) kidney tissue repair mechanisms are, at least partly, mediated *via* inflammatory mediators and TLRs. We hypothesize that also in this maladaptive regeneration phase of AKI, which could lead to the formation of fibrosis, recAP could play a therapeutic role. Dephosphorylation of different PAMPs and DAMPs by recAP could influence the DAMP/PAMP–receptor interaction which results in to less fibrosis formation and therefore positively influence longer-term renal function. We propose that future research will look into this mechanism of recAP in relation to prevention of maladaptive repair mechanisms and fibrosis.

Following these two small phase 2 trials it was decided to develop a human recombinant AP, as biAP retrieved from calf intestine has various drawbacks, including the (minimal) risk of bovine spongiform encephalopathy and occurrence of an undesirable immune response. In the development of the human recombinant form of AP (recAP), it was decided to combine the biologically active intestinal AP and the very stable placental AP. The resultant biologically active and long half-life recAP was produced for the upcoming large phase 2 clinical trial (**Figure 1**)(Kiffer-Moreira et al., 2014).

The STOP-AKI trial was the first international, randomized, double-blind, placebo-controlled trial evaluating the effect of recAP on kidney function during SA-AKI and was designed to determine the optimal dose of recAP and to evaluate adverse events and efficacy (Pickkers et al., 2018). The trail enrolled 301 patients in 11 countries who were diagnosed with SA-AKI. Following blinded interim analysis, the 1.6 mg/kg recAP (once daily on three consecutive days) group was favored to continue the study. The primary endpoint focused on the mechanism of action, an improvement of ECC within 7 days, powered to detect a difference of 19 ml/min in ECC over the first week following enrolment. Unfortunately, despite randomization, the recAP treatment group had a somewhat lower median [interquartile range (IQR)] ECC on day 1 of 26.0 [8.8–59.5] ml/min, compared to the placebo group (35.9 [12.2–82.9] ml/min). Seven days later ECC increased to 65.4 [26.7–115.4] ml/min in the recAP group, compared to 61.9 [22.7–115.2] ml/min in the placebo group, a difference that was not statistically significant. Nevertheless, ECC improvement from enrolment up to day 28 was significantly better in the recAP group compared to the placebo group and this treatment effect was caused by the difference that occurred on day 21 (mean difference, 16.3 ml/min [95% confidence interval (CI), 3.1 to 29.5]; p = 0.02), and day 28 (mean difference, 18.5 ml/min [95%CI, 5.3 to 31.7]; p = 0.006). Furthermore, all-cause mortality at day 28 was 14.4% for the recAP group versus 26.7% for the placebo group (p = 0.02), this difference persisted until the end of follow-up at day 90 (17.1% for the recAP group versus 29.1% for the placebo group; p = 0.03). *Post hoc* exploratory analyses were performed to test the robustness of the observed survival benefit in the recAP group. Forward stepwise multivariable analysis found 15 covariates that are associated with outcome. Treatment with recAP, disease severity score, baseline ECC, and time to recAP treatment emerged as significant independent prognostic factors for survival. The short-term renal outcome discrepancy could be due to a number of factors. First, the estimated kidney function based on ECC is known to be imprecise. Creatinine-based calculations to estimate kidney function are often used but with recognized limitations in the acute phase of critical illness, as serum creatinine is a late and insensitive marker of kidney function. However, a more precise marker of kidney function is currently not available. Additionally, the recAP-treated group of the STOP-AKI trial had a slightly more impaired kidney function upon study initiation, which could account for a less pronounced improvement of ECC in the first week. Analysis of the short-term renal function improvement did, however, not take into consideration the 8–10 ml/min baseline kidney function disadvantage in the recAP-treated group. Exploratory *post hoc* analysis of the STOP-AKI trial found that baseline ECC strongly correlated with renal improvement, suggesting an underestimation of improved kidney function in the recAP-treated group. Considering that the initial degree of kidney dysfunction is prognostic for survival, the significance of the difference in mortality in favor of the recAP-treated group, against this baseline ECC–mortality association, is strengthened by the poorer initial kidney function in this group. The increased survival of the recAP-treated patients might also partly explain the overall moderate improvement of kidney function. It could be argued that, due to recAP treatment, a group of critically ill patients survived SA-AKI that would otherwise have died. Survival of these severely ill patients might have brought down the average kidney function of the recAP-treated group. Conceivably, the 7-day period may have been too short to detect an effect of recAP on kidney function in the larger trial, as differences in creatinine clearance only became clear after 3 and 4 weeks. One might argue that recAP might resolve AKI to a greater extent, compared to influencing the development of AKI. In other words, it appears possible that recAP may modulate the sustained low-grade renal inflammatory response that is associated with the formation of fibrosis and longer-term impaired renal function to a greater extent than interfering in the acute processes associated with the development of AKI in the early phase. This possibility is of interest, but currently, there is no preclinical research available into this putative mechanism of action of AP.

## Discussion

Administration of exogenous AP was initially thought to be of potential therapeutic use as a general antisepsis drug aimed to modulate the systemic inflammatory response by detoxifying PAMPs (such as LPS) and DAMPs (such as extracellular ATP) by dephosphorylation. However, in two small clinical trials it emerged that the therapeutic efficacy on the kidney was most pronounced. Subsequently, the focus shifted toward a specific treatment for SA-AKI. In the large phase 2 trial applying human recombinant AP, no safety issues emerged, but the compound failed to demonstrate a statistically significant difference in primary endpoint: improvement of short-term renal function. Nevertheless, longer-term renal function was beneficially influenced by recAP and, even more impressive, survival was significantly better in patients treated with recAP compared to patients receiving placebo. Following additional analyses, it appears that this survival benefit is robust. As impaired kidney function leads to a series of nonrenal, but potentially life threatening consequences during the later course, e.g., fluid overload causing cardiovascular failure, prolonged weaning from mechanical ventilation, and impaired nutrient absorption as well as drug toxicity due to dosing difficulties (Elapavaluru and Kellum, 2007), the longer-term beneficial effects of recAP on the kidneys may account for the observed survival benefit.

### Mechanism of Action

So far, research related to the therapeutic efficacy of AP has focused on attenuation of the inflammatory response elicited by PAMPs and DAMPs. Based on laboratory and animal data, modulation of these pathways by AP appears to account for the observed beneficial effects of AP administration. Indeed, it seems plausible that these mechanisms of action are also of relevance for the therapeutic efficacy observed in patients. Nevertheless, the observation in the recent phase 2 trial that treatment with recAP results in improvement of renal function, which is most pronounced 3–4 weeks following enrolment, suggests that recAP may also affect renal processes that occur in this subacute phase. As eluded to earlier, following the initial insult, repair processes in the kidney also involve TLR pathways and inadequate repair processes leading to fibrosis are also at least partly mediated through these pathways (Anders and Schaefer, 2014). It is currently unknown whether or not AP is able to interfere in these pathways. If so, recAP-mediated stimulation of regeneration may account for the protective effects observed 21 and 28 days following enrolment, in addition to modulation of acute inflammation-induced damage.

A possible therapeutic effect of recAP on sepsis may lead to general improvements, that may indirectly lead to a better renal function. However, in the STOP-AKI trial the course of organ dysfunction of different organs was followed and organ improvement was most pronounced in the kidneys, suggesting that a renal effect may be present. The positive effects of recAP observed on kidney function after 3 and 4 weeks and on survival in the STOP-AKI trial are undoubtedly promising, and arguably even more important than just early improvement in kidney function. The long half-life of the recombinant AP may be of importance of the observed longer-term renal effects. Consequently, a prospective phase III trial is needed to confirm these results. Apart from survival, if enhanced kidney function turns out to be a sustained effect, this could lead to a decrease in the occurrence of end-stage renal disease following AKI.

Several other issues are of interest to consider. The clinical trials in which the clinical efficacy of AP was investigated were performed in sepsis patients with early AKI. Starting treatment earlier during the course of the illness, e.g., when AKI is not yet diagnosed, could potentially maximize the protective effect of AP and prevent the occurring of damage. In view of the profound effect on survival, lack of safety concerns related to the administration of AP that would argue against a more broad indication, this would be of interest. Similarly, as AP was shown to exert renal protective effects in other preclinical models of AKI [e.g., ischemia–reperfusion (Peters et al., 2016), or cisplatinum-induced (Peters et al., 2014b) AKI], future clinical trials in other patient groups would be of interest as well.

In summary, AP is a detoxifying enzyme that exerts effects by dephosphorylation of various compounds. Exogenous administration of AP exerts kidney protective effects in various animal models of sepsis. Two small phase 2 trials with bovine AP showed beneficial renal effects. A recently completed large phase 2 trial applying a human recombinant AP failed to show a benefit on creatinine clearance during the first 7 days, but improved longer-term renal function and survival. The focus of the renal protection has been on attenuation of the initial renal inflammatory response. The observed beneficial renal effects 3–4 weeks following enrolment may suggest that recAP might also play a role in the modulation of inflammation-mediated repair mechanisms in the kidney. We advocate that future mechanistic studies focus on this putative mechanism of action.

## Author Contributions

All authors equally contributed to the writing of this manuscipt.

## Conflict of Interest Statement

PP received consultancy and travel reimbursements from AM-Pharma as the PI and medical monitor of the STOP-AKI study. The remaining authors declare that the research was conducted in the absence of any commercial of financial relationships that could be constructed as a potential conflict of interest.
